# Fast Identification and Quantification of Graphene Oxide in Aqueous Environment by Raman Spectroscopy

**DOI:** 10.3390/nano10040770

**Published:** 2020-04-16

**Authors:** Shengnan Yang, Qian Chen, Mengyao Shi, Qiangqiang Zhang, Suke Lan, Tusunniyaze Maimaiti, Qun Li, Peng Ouyang, Kexin Tang, Sheng-Tao Yang

**Affiliations:** College of Chemistry and Environment Protection Engineering, Southwest Minzu University, Chengdu 610041, China; yangsn1993@163.com (S.Y.); chenqian2224597@163.com (Q.C.); 18328095288@163.com (M.S.); zqq950526@163.com (Q.Z.); lansukexn@163.com (S.L.); t1737867950@163.com (T.M.); qwe1170954047@163.com (Q.L.); oybridge@163.com (P.O.); kexintang@126.com (K.T.)

**Keywords:** graphene, Raman spectroscopy, chemical reduction, environmental analysis, nano-biosafety

## Abstract

Today, graphene nanomaterials are produced on a large-scale and applied in various areas. The toxicity and hazards of graphene materials have aroused great concerns, in which the detection and quantification of graphene are essential for environmental risk evaluations. In this study, we developed a fast identification and quantification method for graphene oxide (GO) in aqueous environments using Raman spectroscopy. GO was chemically reduced by hydrazine hydrate to form partially reduced GO (PRGO), where the fluorescence from GO was largely reduced, and the Raman signals (G band and D band) were dominating. According to the Raman characteristics, GO was easily be distinguished from other carbon nanomaterials in aqueous environments, such as carbon nanotubes, fullerene and carbon nanoparticles. The GO concentration was quantified in the range of 0.001–0.6 mg/mL with good linearity. Using our technique, we did not find any GO in local water samples. The transport of GO dispersion in quartz sands was successfully quantified. Our results indicated that GO was conveniently quantified by Raman spectroscopy after partial reduction. The potential applications of our technique in the environmental risk evaluations of graphene materials are discussed further.

## 1. Introduction

Graphene and its derivatives are produced on a large-scale today, because graphene materials have a unique structure and fantastic properties [1,2]. Graphene has been widely applied in electronics [3], optical applications [4], catalysis [5], biomedicine [6], analysis [7], environmental technologies [8], and so on. For instance, graphene adsorbents have been well developed to remove Cu^2+^ from water and soil samples, which reduced the Cu^2+^ bioaccumulation in wheat seedlings [9]. Graphene-ZnO composite showed strong antibacterial activity for disinfection applications [10]. Graphene-Fe_3_O_4_-TiO_2_ composite catalyzed the Fenton reaction to generate hydroxyl radicals with good recycling properties [11]. With the success of graphene, many production lines have been built to produce hundreds of tons of graphene every year. Furthermore, the environmental risks and toxicity of graphene have aroused great concerns [12,13,14]. 

The literature results collectively indicated that graphene was toxic to environmental organisms [12,13,14]. GO induced the damage to wheat roots through oxidative stress [15]. GO also inhibited white rot fungus growth, disturbed the structure of mycelium, and decreased the decomposition activity [16]. GO led to the cell death, growth inhibition, cell membrane damage, and cytoplasm loss of nitrogen-fixing bacteria [17]. GO induced meaningful toxicity to mice after an intravenous injection and pulmonary exposure [18,19]. During these studies, GO showed obvious concentration-dependent toxicity. Therefore, the environmental concentration of graphene should be carefully quantified to estimate the toxicity.

The identification and quantification of graphene is the first step to evaluating the environmental risk and hazards. There are two major techniques for the quantification of graphene. Firstly, graphene can be analyzed by isotope labeling [15,20,21,22]. We developed a ^13^C-skeleton labeling technique for the quantification of GO and reduced GO (RGO) in plants [15,20]. ^13^C-GO and ^13^C-RGO induced the isotope ratio changes in plants and could be detected by isotope ratio mass spectroscopy. Mao et al. quantified ^14^C-graphene in mice, plants and aquatic species [22]. Secondly, dispersible graphene materials (mainly GO) could be quantified by a UV-Vis spectrometer following the Lambert-Beer law [23,24]. Such attempts were widely reported in diverse applications and transport studies of GO [25,26]. However, isotope labeling is not suitable for practical samples, because the graphene materials were not isotope labeled during the manufactured production. Spectrophotometry could not distinguish the absorbance of GO from that of other substances, leading to false positive data. Thus, new facile methods are highly in demand for the quantification of graphene in environmental samples.

Raman spectroscopy is a well-established technique for characterizing graphene, which has the unique signals G band and D band [27]. Raman spectroscopy has been adopted in the quantification of carbon nanotubes (CNTs) in biological samples [28,29]. For example, Liu et al. used Raman spectroscopy to quantify the pharmacokinetics and biodistribution of poly(ethylene glycol) modified CNTs in mice [29]. However, Raman spectroscopy is not suitable in the quantification of GO, because GO has strong fluorescence interference [30]. Therefore, it is necessary to develop a facile technique to reduce the fluorescence of GO for Raman spectroscopic quantification.

In this study, we reduced GO by hydrazine hydrate to form partially reduced GO (PRGO), and achieved the quantification of GO in an aqueous environment using Raman spectroscopy, where hydrazine hydrate has been well proven as an efficient reducing reagent for GO [31]. GO and PRGO were carefully characterized to explain the mechanism of fluorescence quenching. The identification of different carbon nanomaterials in an aqueous environment using Raman spectroscopy was established and the local water samples from Chengdu city were analyzed. The Raman spectroscopic quantification of GO was used in this study of the transport behaviors of GO in quartz sands. The implications of the Raman spectroscopic quantification of GO on the environmental risk evaluations of graphene materials are discussed further.

## 2. Materials and Methods

### 2.1. Materials

Graphite (99% purity) was obtained from the Shanghai Huayi Group Huayuan Chemical Co., Ltd. All chemicals were of an analytical grade and used without further purification. Ti slides (76 × 26 mm, with three grooves) were bought from Long Jin Metal Co. (Shanghai, China). GO was prepared by the modified Hummers method. The details can be found in our previous report [32]. To prepare partially reduced GO (PRGO), 1.0 ml of GO solution (0.1 mg/mL) was added to 50 μL of hydrazine hydrate and sonicated for 30 min. After removing the remaining N_2_H_4_·H_2_O by centrifugation, the PRGO was washed with water and dried. Both GO and PRGO were characterized by transmission electron microscopy (TEM, Tecnai G2 20, FEI, Hillsboro, OR, USA), infrared spectroscopy (IR, Magna-IR 750, Nicolet, Madison, WI, USA), X-ray photoelectron spectroscopy (XPS, ESCALAB 250XI, Thermo-Fisher, Waltham, MA, USA) and Raman spectroscopy (inVia, Renishaw, London, UK).

### 2.2. Quantification of GO in Water

Raman analyses of GO dispersion were performed using the protocol described as follows. GO was diluted with water to concentrations of 0.6, 0.4, 0.2, 0.1, 0.08, 0.06, 0.04, 0.02, 0.01, 0.008, 0.006, 0.004, 0.002 and 0.001 mg/mL for calibration. The concentration range of 0.001–0.6 mg/mL was selected because there was no linearity out of that range in our pre-experiments of 0.00001–1.0 mg/mL. Each 1.0 mL of GO dispersion was added to 50 μL of N_2_H_4_·H_2_O, and sonicated for 30 minutes before the Raman analysis. To a groove of a Ti slide, 50 μL of the PRGO dispersion was added. The Raman analyses used the following parameters: method = mapping review, z = −500, excitation wavelength = 532 nm, power = 10%, time = 1 s, scanning wavelength = 700–2400 nm. The data were processed using the signal to baseline method. The average mean and standard derivation (mean ± SD) were obtained. The log(GO concentration) was plotted with Raman intensity by linear fitting. GO dispersions of 0.001, 0.003, 0.005, 0.007, 0.03, 0.05, 0.07 and 0.25 mg/mL were tested using the same protocol to calculate the recovery efficiency.

### 2.3. Analyses of Local Water Samples

Local water samples were collected from eight different spots in Chengdu city, Sichuan province, China (Table 1). The water was analyzed by the established protocol as previously mentioned, with a small change. The natural water contained large quantities of Ca^2+^ and Mg^2+^, which would lead to the aggregation of GO during the reduction, so each water sample (1 mL) was added to 50 μL of EDTA (2 mg/mL) before the addition of N_2_H_4_·H_2_O.

### 2.4. Transport of GO in Quartz Sand Column

To investigate the transport behaviors of GO in a quartz sand column, the quartz sand was carefully washed with water and dried at 60 °C overnight before use. The sand was packed into a glass column (diameter of 1.5 cm, length of 12 cm) and dampened with water. The column was operated in a downward direction. After the equilibrium with the water, the water was replaced with the GO dispersion (0.25 mg/mL) as the mobile phase. The eluent was collected at 2 mL each, for GO concentration determination following the protocol in Section 2.2. After reaching the plateau, the mobile phase was replaced with water.

For comparison, the samples were also analyzed by a UV-Vis spectrometer (UV1600, Shanghai Mapada Instruments Co., Shanghai, China). First, the absorbance of GO dispersions at different concentrations was measured at 400 nm, to build the calibration curve. Then, the absorbance of transport experiment samples was measured at 400 nm, and the GO concentrations were calculated for the calibration curve.

## 3. Results and Discussion

### 3.1. Characterization of GO and PRGO

**Figure 1:**
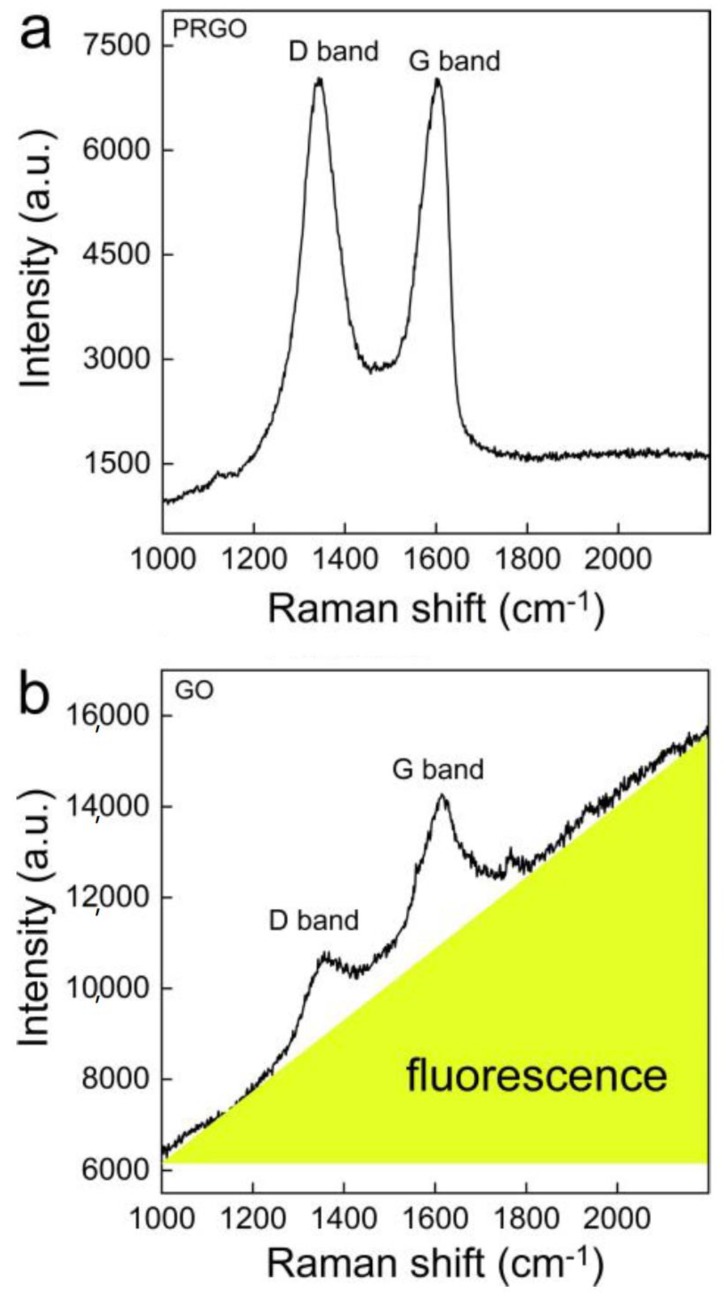
Raman spectra of partially reduced GO (PRGO) (**a**) and graphene oxide (GO) (**b**). The GO suspension had strong fluorescence as the background (highlighted in yellow).

Typically, GO fluorescence is due to electron-hole recombination from the bottom of the conduction band and nearby localized electronic states to the wide-range valance band [33]. GO emission is predominantly from the electron transitions among/between the unoxidized carbon region (C=C) and the boundary of the oxidized carbon region (C–O, C=O and O=C–OH). The 785 nm excitation could largely reduce the fluorescence but would simultaneously largely decrease the Raman intensity. Alternatively, the reduction in GO was supposed to decrease the fluorescence interference and retain the Raman signals. To verify this, we recorded the raw Raman spectra of both GO and PRGO (Figure 1). The sharp peaks of the D band and G band could easily be recognized in the PRGO. The background was low and generally flat. In contrast, without a reduction there was a strong fluorescence background in the Raman spectrum of GO. The D band and G band peaks were weak and broad. In addition, the *I*_D_/*I*_G_ increased after the reduction, which was also widely observed in the literature [34]. Therefore, the Raman analyses confirmed that a chemical reduction could improve the signal to noise ratio.

**Figure 2:**
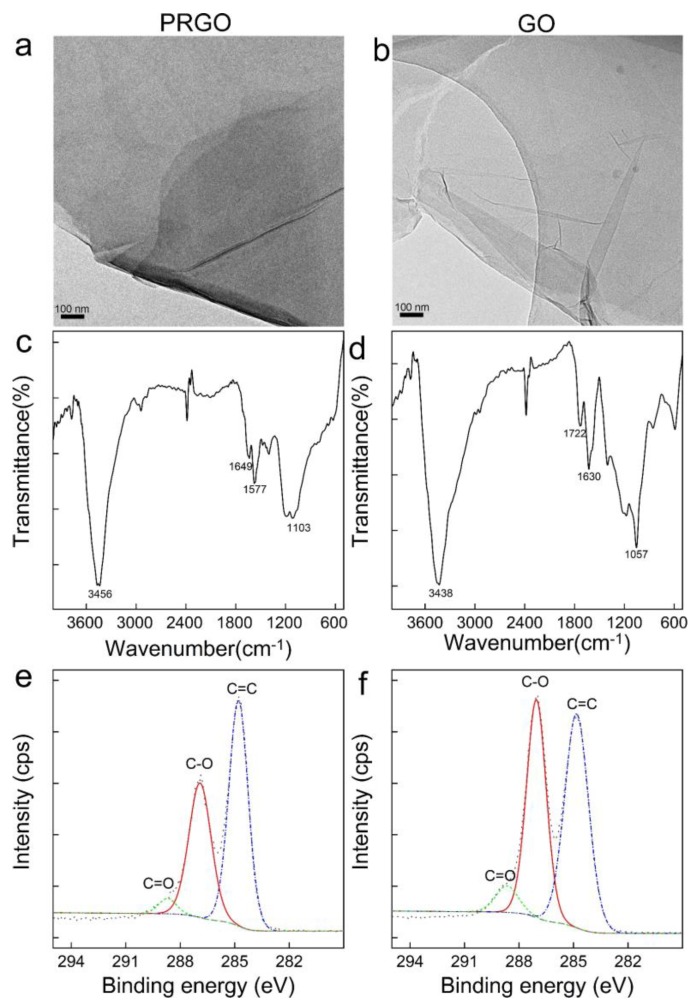
Characterizations of PRGO and GO. (**a**) TEM image of PRGO; (**b**) TEM image of GO; (**c**) IR spectrum of PRGO; (**d**) IR spectrum of GO; (**e**) C1s XPS spectrum of PRGO; (**f**) C1s XPS spectrum of GO.

PRGO and GO were further characterized to prove the hypothesis (Figure 2). In the TEM images, both PRGO and GO showed sheet-like structures, while graphene sheets were stacked more intensely in PRGO (higher contrast). The IR spectra showed very similar characteristics, because PRGO still had oxygen containing groups. According to the literature, the 1649 cm^−1^ band of PRGO and 1630 cm^−1^ band of GO was assigned to unoxidized graphitic domains [35]. However, the C=O stretching vibration of GO at 1722 cm^−1^ could not be found in the PRGO, suggesting the efficient reduction. Similarly, the C–O (alkoxy) stretching vibration at 1057 cm^−1^ decreased sharply after reduction. The peak at 1412 cm^−1^ of GO or 1404 cm^−1^ of PRGO was attributed to O–H deformation. The peak at 1212 cm^−1^ was assigned to the C–O (epoxy) stretching vibration. In the C1s XPS spectra, PRGO had more C–C (57.0% of C–C, 39.4% of C–O and 3.5% of C=O), while GO had C–C (51.2%), C–O (42.9%) and C=O (5.9%). The elemental compositions were 68.9% of C, 5.2% of N and 25.9% of O for PRGO, while the numbers were 68.4% of C, 1.3% of N and 30.3% of C for GO. The characterization data suggested that the reduction lowered the oxygen content of GO and kept the sheet-like structure. According to the literature, the oxygen-enriched defects were the main contributor to fluorescence [30,33], thus the decrease in fluorescence after reduction was reasonable.

### 3.2. Quantification of GO in Water

**Figure 3:**
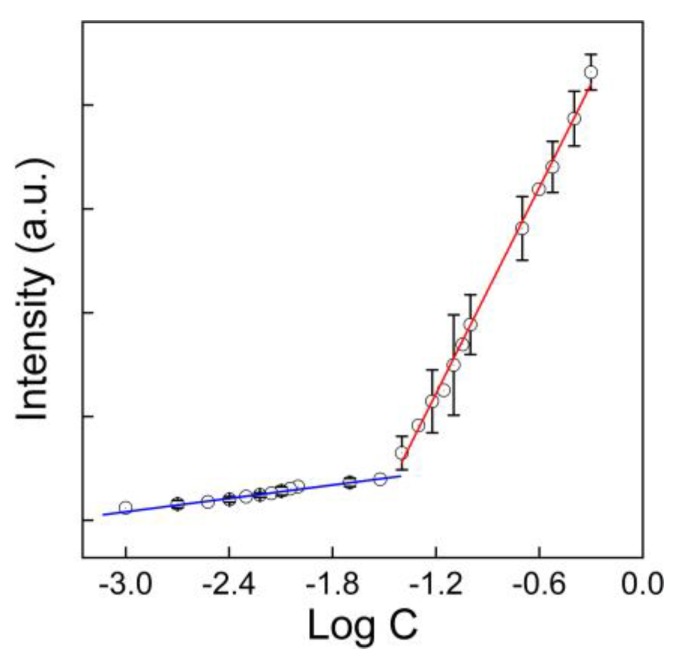
Calibration lines as G band intensity versus log(GO concentration).

To establish the quantification method for GO, we prepared the GO dispersions of different concentrations and reduced them before the Raman analyses. Due to the reduction, the PRGO was slightly aggregated into small particulates, making the system heterogeneous. To reduce the error from heterogeneity, a mapping model was adopted to collect over 100 datum points for each sample and three replicates were measured for each concentration. There are characteristic D, G, 2D and D + D’ bands for GO [36]; however, the G band was the strongest one. Subsequently, we adopted the G band for the quantification. As shown in Figure 3, the intensity of the G band showed good linearity to the logarithmic concentration of GO in two different concentration ranges. In 0.01–0.6 mg/mL, the G band intensity followed Equation (1) with *R*^2^ of 0.995. In 0.001–0.1 mg/mL, the G band intensity followed Equation (2) with *R*^2^ of 0.966. The recovery efficiencies were measured and are listed in Table 2. The Raman quantification was more accurate in the higher concentration range of 0.01–0.6 mg/mL. In the lower concentration range, the aggregates were fewer and the total intensity was smaller, resulting in lower recovery efficiencies. The samples with concentrations higher than 0.6 mg/mL could be easily diluted and quantified with our method. However, for those samples with concentrations lower than 0.001 mg/mL, they could not be concentrated into the linear range, because serious aggregation during the concentrating process occurred.

*y* = 162324*x* + 257022 (1)

*y* = 8730*x* + 31688 (2)

**Figure 4:**
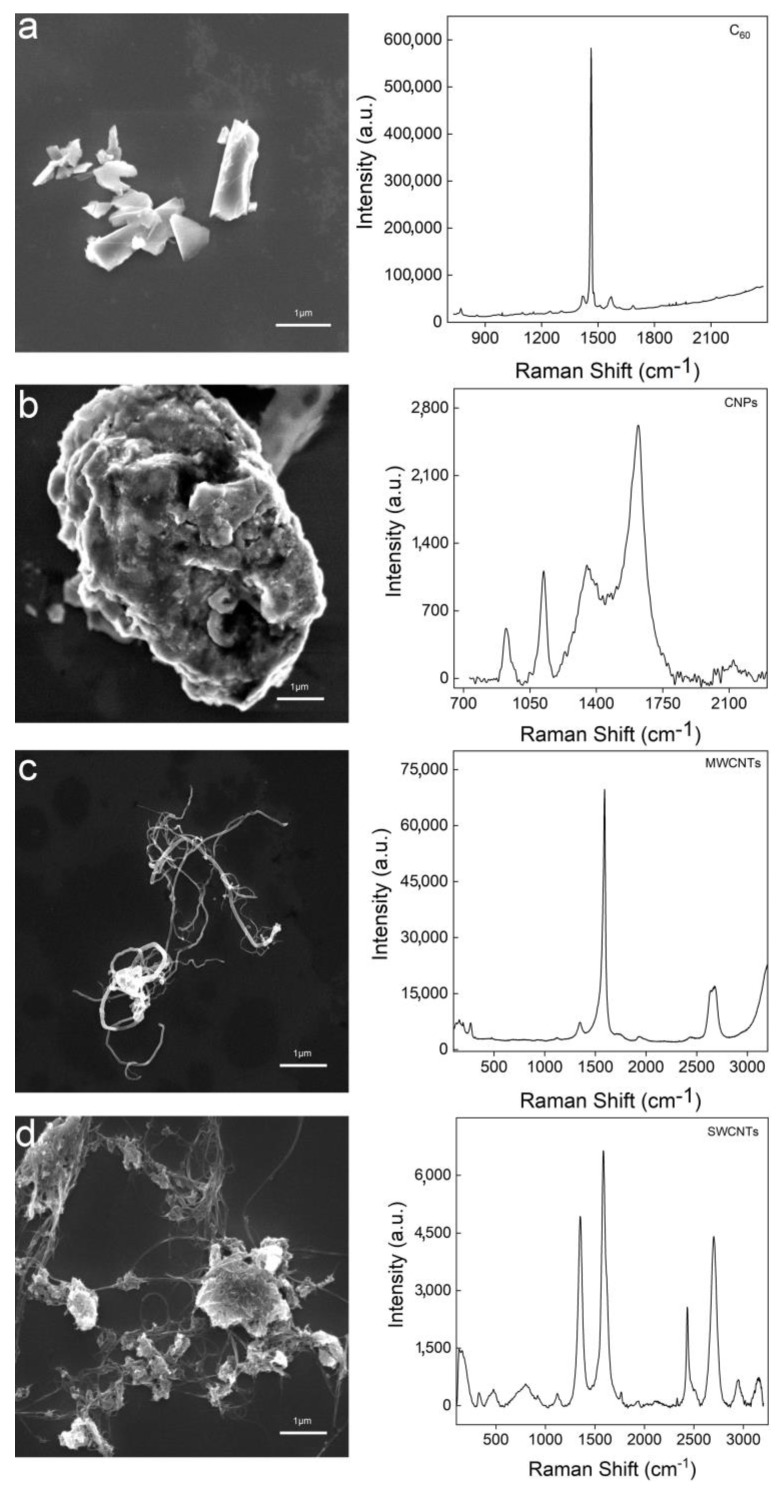
Identification of different carbon nanomaterials in aqueous environment by Raman spectroscopy with the assistance of SEM. (**a**) fullerene C_60_; (**b**) Carbon nanoparticles (CNPs); (**c**) Multi-walled CNTs (MWCNTs); (**d**) Single-walled CNTs (SWCNTs). Left panels are the SEM images and the right panels are the Raman spectra.

One advantage of the Raman quantification over spectrophotometry was that the Raman spectrum could be used to distinguish GO from other carbon nanomaterials. In the UV-Vis spectroscopy, it was hard to identify the specie of carbon nanomaterial. To verify this, we prepared the aqueous dispersions of different carbon nanomaterials and characterized them by scanning electron microscopy (SEM) and Raman spectroscopy. In Figure 4, fullerene was sharp blocks and the characteristic Raman peak showed at 1466 cm^−1^. Carbon nanoparticles (CNPs) wrapped into large aggregates. Their D band and G band crowded together and the peaks were broad. Multi-walled CNTs (MWCNTs) showed a fibrous structure under SEM. The G band was very sharp, but the D band was very small due to the lack of defects. Single-walled CNTs (SWCNTs) similarly showed a fibrous structure. There were D band and G bands in the Raman spectrum of SWCNTs, but the intense 2D band was quite different from that of the PRGO. Therefore, using Raman spectroscopy, we could easily identify GO from other carbon nanomaterials in aqueous samples, which could further be confirmed by checking under SEM. It is worthwhile to note that our GO was epoxy-rich GO, which needs to be distinguished from carboxylic rich GO in the future [37]. The interference study with other carbon materials or different carbon sources for GO, the effect of metal ions loading, the effect of delamination behavior and the effect of the redundant ratio should also be optimized for the future.

### 3.3. Analyses of Local Water Samples

**Figure 5:**
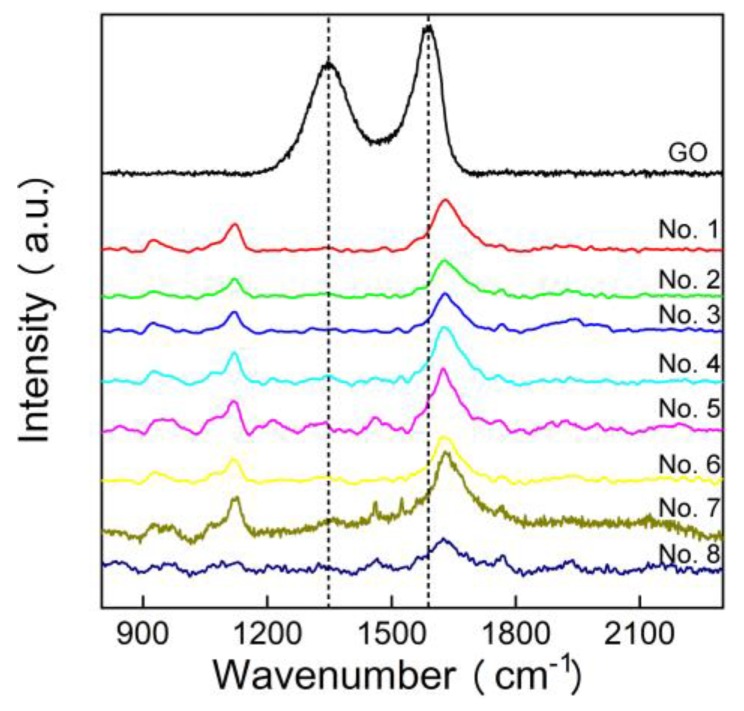
Raman spectra of local water samples after reduction.

The first application of our method was to analyze the local water samples. We collected eight different water samples in Chengdu city (Table 1). The water samples were treated and measured following our protocols. In Figure 5, there was no meaningful graphene signal detected among all eight samples. There was no G band or D band peak observed in those spectra. The peak at 1620 cm^−1^ was attributed to water or excess hydrazine hydrate (Figure S1). This suggested that there was no graphene or GO in the aqueous environment, although Chengdu has been dedicated to developing a graphene industry for years. The absence of graphene in the aqueous environment indicated that graphene has not yet induced pollution in Chengdu city.

### 3.4. Transport of GO in Quartz Sands

**Figure 6:**
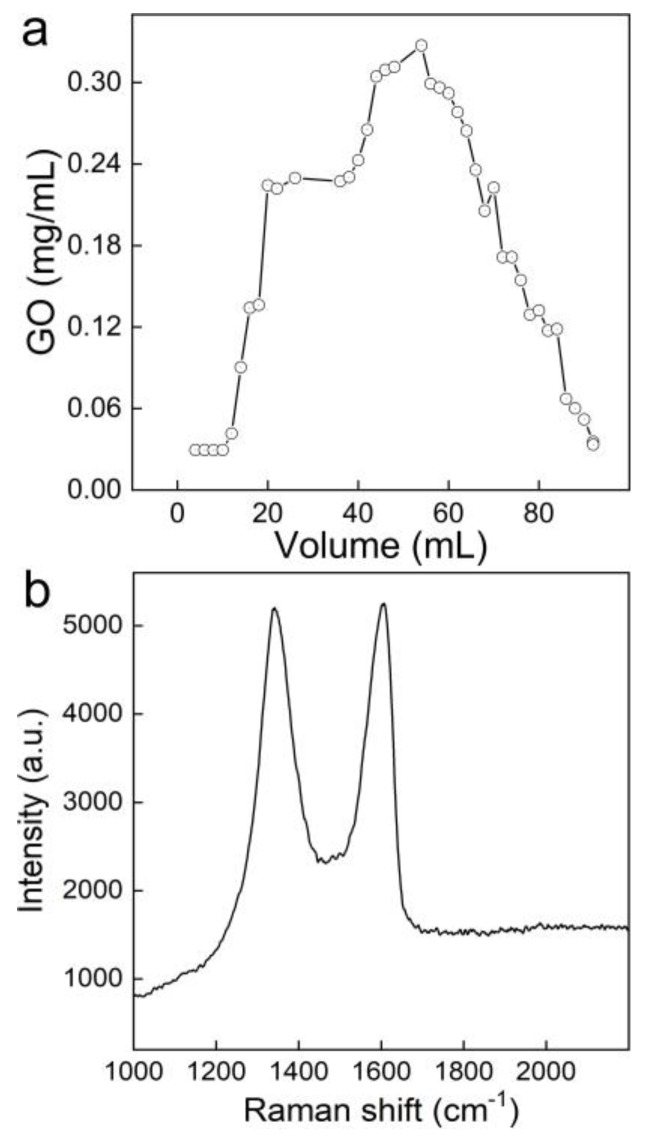
Transport of GO through a quartz sand column. (**a**) GO concentrations in eluents; (**b**) a representative Raman spectrum of the eluent samples containing GO.

The second application of our method was to analyze the transport behaviors of GO in the quartz sands. As shown in Figure 6a, initially GO was adsorbed in quartz sands, which resulted in no GO being detected in the eluent. After the addition of 15 mL of the GO dispersion, the quartz sands were saturated and the GO concentrations in the eluent increased gradually to reach the plateau. The characteristic G band and D band signals of GO were presented in each single spectrum during the mapping model detection (Figure 6b), suggesting the existence of GO in the eluent. Furthermore, the eluent also showed the brown color of GO. After replacing the mobile phase by deionized water, the GO concentration in the eluent decreased. The unadsorbed GO was washed out. However, the sands kept the brown color, suggesting that there were graphene sheets trapped by the sands, even after the eluent concentration became 0 mg/mL.

**Figure 7:**
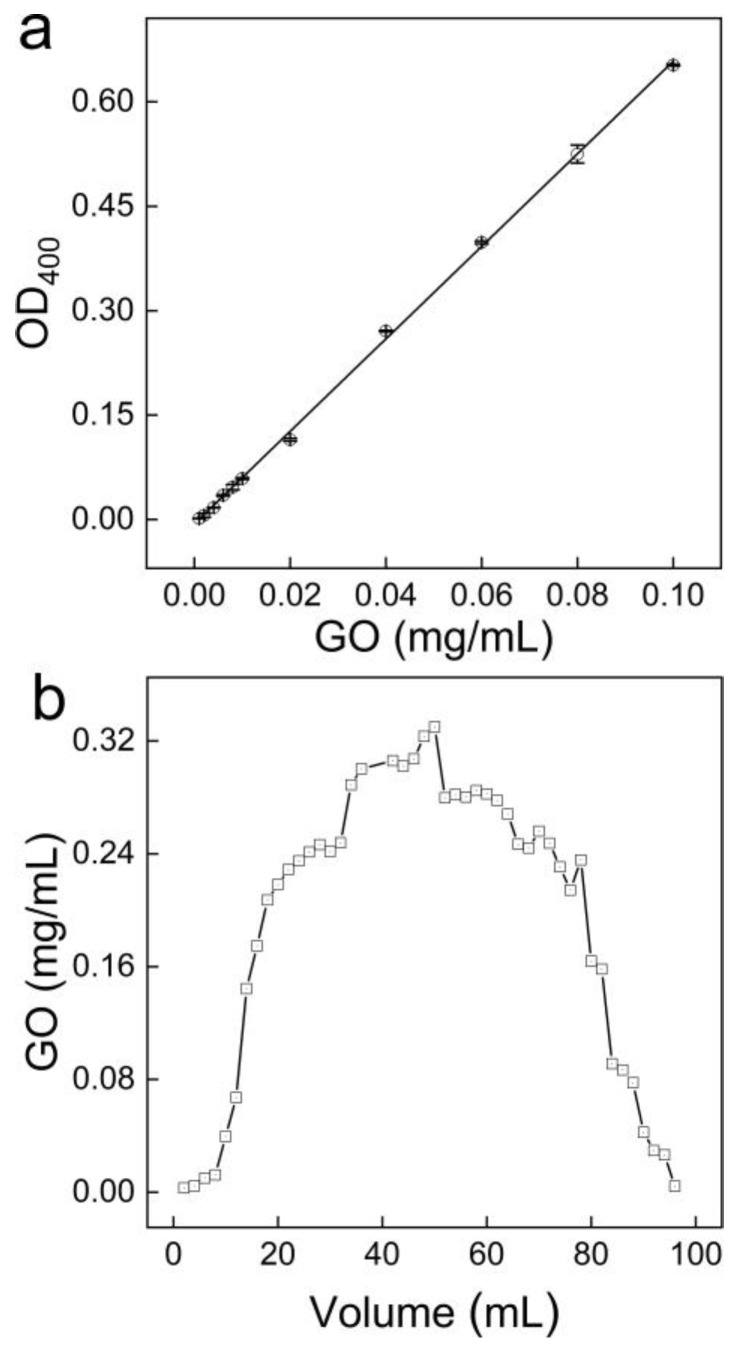
Transport of GO through a quartz sand column analyzed by spectrophotometry. (**a**) the calibration line; (**b**) GO concentrations in eluents. The detection wavelength was set at 400 nm.

To further confirm the reliability of our method, we also achieved the transport evaluation using UV-Vis spectroscopy. In Figure 7, we first established the calibration curve showing the linearity range of 0.001–0.1 mg/mL, which was consistent with the literature results [23]. At concentrations lower than 0.001 mg/mL, the absorbance was too small for efficient detection. The transport behavior of GO was very similar to that obtained by our Raman method, indicating that our Raman method was reliable. In future studies, our Raman method could be applied in analyzing the transport behavior of GO in the presence of colored molecules, e.g., dyes and humic acid, that would interfere with the UV-vis spectroscopic quantification. For example, the Raman signal of GO could be easily distinguished from that of humic acid (Figure S2).

## 4. Conclusions

In summary, GO was quantified by Raman spectroscopy after the partial reduction by N_2_H_4_·H_2_O, where the reduction largely decreased the fluorescence interference from GO defects. GO could be quantified in water in the concentration range of 0.001–0.6 mg/mL. The signal of GO could be easily distinguished from those of other carbon nanomaterials. The established technique was successfully applied in analyses of the local water samples and the transport of GO dispersion in a quartz sand column. It is hoped that the Raman quantification of GO will benefit environmental impact evaluations and the safe applications of graphene nanomaterials.

## Figures and Tables

**Table 1:** Local water samples from Chengdu city.

Sample	Location	Latitude	Longitude
No. 1	Wanli bridge	30.64723	104.06004
No. 2	Lotus pond of Sichuan University	30.63913	104.06933
No. 3	Wenshu monastery	30.67574	104.07183
No. 4	Culture park	30.6605	104.0431
No. 5	Drunk plum garden	30.65801	104.04321
No. 6	Baihuatan park	30.65612	104.04365
No. 7	Library of Southwest Minzu University	30.63849	104.04742
No. 8	Rain in Southwest Minzu University	30.63849	104.04742

**Table 2:** Recoveries of the spiked water samples.

Spiked GO (mg/mL)	Determined GO (mg/mL)	Recovery (%)
0.25	0.2505 ± 0.0051	100.2%
0.07	0.0634 ± 0.0065	90.6%
0.05	0.0499 ± 0.0064	99.7%
0.03	0.0345 ± 0.0029	115.1%
0.007	0.0072 ± 0.0071	103.2%
0.005	0.0047 ± 0.0003	94.6%
0.003	0.0024 ± 0.0004	80.8%
0.001	0.0011 ± 0.0001	114.3%

## Supplementary Materials

The following are available online at https://www.mdpi.com/2079-4991/10/4/770/s1, Figure S1: Raman spectra of hydrazine hydrate (a), water (b) and hydrazine hydrate + water (c)., Figure S2: Raman spectra of GO+tannic acid (top) and tannic acid (bottom).
