# Supplementary material for: Fast Identification and Quantification of Graphene Oxide in Aqueous Environment by Raman Spectroscopy
Source: Nanomaterials (Basel). 2020 Apr 16;10(4):770. doi: 10.3390/nano10040770 (PMC7221548; doi:10.3390/nano10040770)
Supplement: Supplementary file 1 [file nanomaterials-10-00770-s001.pdf]

Supplementary materials

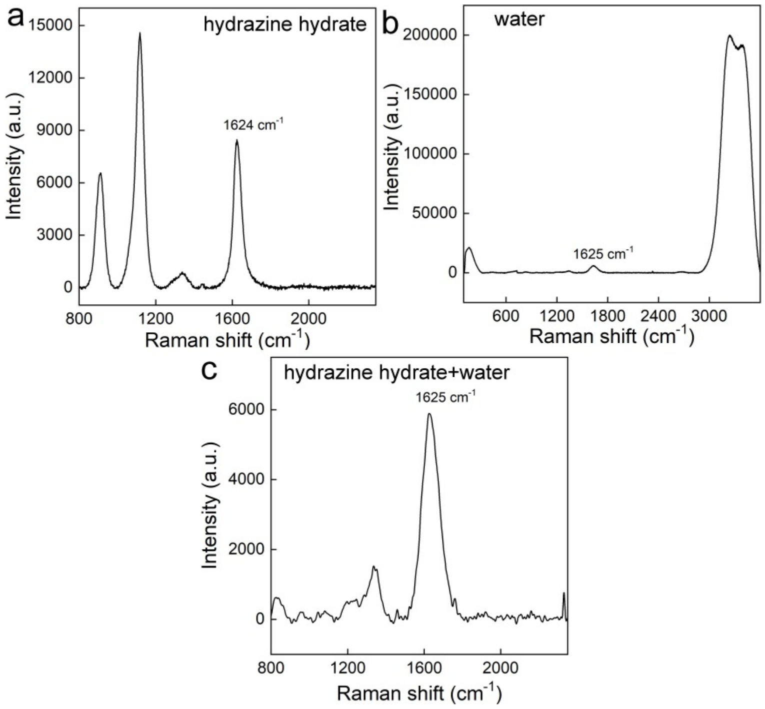

Figure S1. Raman spectra of hydrazine hydrate (a), water (b) and hydrazine hydrate+water (c).

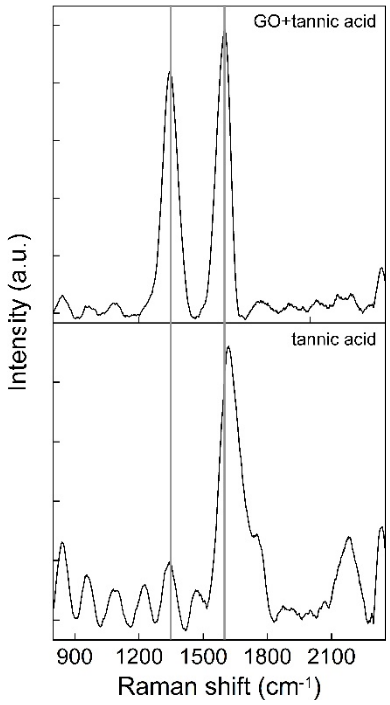

Figure S2. Raman spectra of GO+tannic acid (top) and tannic acid (bottom).
